# Polysaccharides and Their Derivatives as Potential Antiviral Molecules

**DOI:** 10.3390/v14020426

**Published:** 2022-02-18

**Authors:** Hadrien Claus-Desbonnet, Elsa Nikly, Vanya Nalbantova, Diana Karcheva-Bahchevanska, Stanislava Ivanova, Guillaume Pierre, Niko Benbassat, Plamen Katsarov, Philippe Michaud, Paolina Lukova, Cédric Delattre

**Affiliations:** 1 Université Clermont Auvergne, CNRS, Clermont Auvergne INP, Institut Pascal, F-63000 Clermont-Ferrand, France; hadrien.claus-desbonnet@etu.uca.fr (H.C.-D.); elsa.nikly@etu.uca.fr (E.N.); guillaume.pierre@uca.fr (G.P.); philippe.michaud@uca.fr (P.M.); 2 Department of Pharmacognosy and Pharmaceutical Chemistry, Faculty of Pharmacy, Medical University-Plovdiv, 4002 Plovdiv, Bulgaria; vanya.nalbantova@mu-plovdiv.bg (V.N.); diana.karcheva@mu-plovdiv.bg (D.K.-B.); benbassatnicko@abv.bg (N.B.); paolina.lukova@mu-plovdiv.bg (P.L.); 3 Department of Pharmacognosy and Pharmaceutical Botany, Faculty of Pharmacy, Medical University Sofia, 1000 Sofia, Bulgaria; 4 Department of Pharmaceutical Sciences, Faculty of Pharmacy, Medical University-Plovdiv, 4002 Plovdiv, Bulgaria; plamen.katsarov@mu-plovdiv.bg; 5 Research Institute, Medical University-Plovdiv, 4002 Plovdiv, Bulgaria; 6 Institut Universitaire de France (IUF), 1 Rue Descartes, 75005 Paris, France

**Keywords:** polysaccharides, antiviral activities, virus and coronavirus, severe acute respiratory syndrome, SARS-CoV, replication inhibition, side effects

## Abstract

In the current context of the COVID-19 pandemic, it appears that our scientific resources and the medical community are not sufficiently developed to combat rapid viral spread all over the world. A number of viruses causing epidemics have already disseminated across the world in the last few years, such as the dengue or chinkungunya virus, the Ebola virus, and other coronavirus families such as Middle East respiratory syndrome (MERS-CoV) and severe acute respiratory syndrome (SARS-CoV). The outbreaks of these infectious diseases have demonstrated the difficulty of treating an epidemic before the creation of vaccine. Different antiviral drugs already exist. However, several of them cause side effects or have lost their efficiency because of virus mutations. It is essential to develop new antiviral strategies, but ones that rely on more natural compounds to decrease the secondary effects. Polysaccharides, which have come to be known in recent years for their medicinal properties, including antiviral activities, are an excellent alternative. They are essential for the metabolism of plants, microorganisms, and animals, and are directly extractible. Polysaccharides have attracted more and more attention due to their therapeutic properties, low toxicity, and availability, and seem to be attractive candidates as antiviral drugs of tomorrow.

## 1. Introduction

Viruses are parasites that contain an RNA or DNA genome surrounded by a protective, virus-coded protein coat [1,2]. Viruses are considered not only one of the most abundant biological entities on Earth [3] but also one of the major causes of mortality, and, at the same time, they are the driver of genetic diversity on our planet [4]. These parasites can be found everywhere in our surroundings (including in water and soil) and can infect all life forms [5,6,7,8,9,10,11].

Humanity has survived many severe pandemics previously [12,13,14]. In the last four decades, humankind has faced several deadly viral outbreaks, such as human immunodeficiency virus (HIV), severe acute respiratory syndrome coronavirus (SARS-CoV), H1N1 influenza virus, Middle East respiratory syndrome coronavirus (MERS-CoV), Ebola, and severe acute respiratory syndrome coronavirus-2 (SARS-CoV-2) [12,14,15,16,17].

The first evidence of a viral epidemic was described in the tenth century in the treatise of Abu Becr (Rhazes) on measles and smallpox. Several mechanisms of viral epidemiology (such as the contagious effect) came to be understood in the next few centuries. However, the discovery of the virus in its current form did not take place until the 19th century [18]. This discovery was due to the research on tobacco mosaic disease by the German scientist Adolf Mayer and the microbiologist Martinus Beijerinck in 1876 [19]. They concluded that the infectious agent of the tobacco plant was not a toxin or an enzyme but a microorganism that was able to reproduce itself. Until the 1930s, the modes of infection and reproduction of the virus were unknown, although the physical aspect of the virus came to be identified to a greater degree. Thanks to the invention of the electron microscope and the development of new techniques to cultivate and produce viruses, the real study of the virus has become possible [20,21]. Since the discovery of the first antiviral compound in the 1950s [22,23], numerous molecules have been synthesized and studied. However, there are many obstacles that must be surmounted before an antiviral molecule can be commercialized. Like all medicines, antivirals must meet different criteria, including having a low toxicity toward the host cell and organism. A real challenge for researchers is to find a molecule that inhibits the virus but, at the same time, has few side effects. Though more than sixty different chemical antiviral compounds exist, half of them have lost their efficiency due to virus mutation and adaptation. The emergence of new pathogenic viruses and especially the current pandemic have highlighted the necessity of the discovery of new antiviral compounds [24].

Many studies have shown that polysaccharides have antiviral activity and, at the same time, low toxicity. These carbohydrates are natural compounds, primary metabolites essential for the growth, development, and reproduction of plants and microorganisms. They have different biological functions, such as structural support, cell recognition, and energy storage. They can be extracted directly from animal tissues, plants, algae, mushrooms, or microorganisms. Different types of polysaccharides are defined according to their sources, including plant, animal, fungal, and microbial polysaccharides. Some of them provide a pharmacological activity that is different from that of the antiviral. For example, heparin is an important medicine that is used as an anticoagulant [25,26]. Researchers have proven that the antiviral activity of polysaccharides is associated with their anionic groups, and particularly with their sulfate groups [27,28,29]. Chemical and/or enzymatic modifications are able to improve their biological activities, leading to the formation of another polysaccharide category, i.e., semisynthetic polysaccharides. The structure and degree of monosaccharide sulfation play a central role in their antiviral activity. According to their structural features, they inhibit the virus cycle at different stages, such as at the internalization, uncoating, and transcription phases, or even by directly killing the virus. Due to their bioavailability, biological activity, and low toxicity, polysaccharides seem to be the molecules of choice for developing new antiviral drugs essential for the future [27,28,29].

## 2. Commercial Antiviral Compounds

Some viral infections have been successfully eradicated by massive vaccination campaigns. For example, smallpox was successfully eradicated due to vaccination [30,31]. Nevertheless, vaccines are not effective against all viruses, and it is necessary to find another treatment to fight infections. For example, humanity still needs a vaccine for the immunodeficiency virus (HIV) and other viral infections.

### 2.1. History of Antiviral Drugs

There are two main approaches to antiviral drug development. The first targets the viruses themselves, and the second targets host cell factors. Antiviral drugs that directly target viruses include inhibitors of virus attachment and virus entry, uncoating inhibitors, polymerase and protease inhibitors, nucleoside and nucleotide reverse transcriptase inhibitors, and integrase inhibitors [32]. Additional difficulties are faced by molecules that influence the internal steps of the viral cycle, as they need to penetrate into the cell in order to be effective. Other potential targets are the essential cellular proteins of the viral cycle [33].

In the beginning of the antiviral era, antiviral molecules were often discovered without knowing their specific targets or their exact mechanisms of action. For example, 5-iodo-2′-desoxyuridine (IDU), an analogue of thymidine [34], was first synthetized as a potential antitumor agent. The first data concerning 5-iodo-2′-desoxyuridine were published in 1959. Further studies associated IDU with other pharmacological effects. IDU was found to be a specific inhibitor of certain DNA viruses, most notably the herpes simplex virus. It became the first antiviral drug to be commercialized. IDU was used in the topical treatment of herpetic eye infection. It can only be used topically because of its cytotoxicity [35]. In fact, IDU was not the first molecule possessing antiviral properties discovered by chance. In 1950 and 1951, Harme et al. described the thiosemicarbazones, an analogue of a semicarbazone but with a sulfur atom instead of an oxygen atom. This molecule had an effect against active *poxvirus vaccinia* virus in mouse and chick embryos [36,37]. Methisazone (*N*-methylisatin-*β*-thio-semi-carbazone, Marboran) was synthetized from the former compound and used in the 1960s for prophylaxis and the treatment of the disease caused by the variola virus, smallpox [38]. Acyclovir is another antiviral drug that was not primarily developed for the treatment of viral infections. It was tested to determine its antiviral properties in 1977 [39]. This compound is, in fact, the substrate of the thymidine kinase, which is encoded by herpes simplex virus (HSV). Once the acyclovir has been phosphorylated by the thymidine kinase, it can be phosphorylated twice by the cellular kinase, resulting in acyclovir triphosphate. This phosphate compound is an inhibitor of viral DNA synthesis. Acyclovir triphosphate is a competitor of deoxyguanosine triphosphate as a substrate for viral DNA polymerase [40]. With almost no adverse effects, this drug became one of the safest drugs to be commercialized.

Today, six decades after the discovery of the first antiviral agent, more than 50 antiviral medicines have been created. Most of these molecules are involved in the treatment of HIV, while others are used against the herpesvirus (HSV: herpes simplex virus; VZV: varicella-zoster virus; CMV: cytomegalovirus), hepatitis B virus (HBV), hepatitis C (HCV), and influenza virus infections [41].

Most antiviral drugs are nucleoside analogues, which permit competition inhibition during the replication of the virus genome.

### 2.2. Development of Antiviral Drugs

Molecules used as antiviral drugs must target some stage of the replication of the virus cycle and also cover several important criteria.

Antiviral drugs must meet the same requirements for ADME properties (absorption, distribution, metabolism, and excretion) as any other therapeutic substance. The molecules must have a low molecular weight, good solubility, easy administration, few side effects, easy and inexpensive production, and rapid elimination from the body. It has been estimated that 40% of therapeutic substance candidates have failed because of their serious adverse effects [42]. Having a low cost of production and commercialization is of key importance, since a lot of viral diseases are present in developing countries [43]. For instance, 75% of deaths from HIV occur in this region of the world, and treatments must be affordable for most of the population there [44].

There are many compounds with in vitro antiviral activity, but most of them also affect host cell functions and have a low therapeutic effect/toxicity ratio for humans, so they never reach the market [45].

It is essential for an antiviral substance to obtain a high therapeutic index. This index is defined by the amount the substance inhibits the vital functions of cells and the amount it blocks viral multiplication. Antiviral chemotherapy is complicated by three factors. Firstly, the antiviral drug must not interfere with the normal cellular metabolism. Secondly, antiviral therapy is not able to eradicate viral infection in latency. Finally, the virus is genetically variable—in other words, viruses can mutate and become resistant to antivirus treatment [46,47].

This resistance is the direct consequence of the genetic variability of the virus and the specificity action of the antiviral drugs employed. The selection of resistant mutants can lead to treatment failure [48]. It is important to have a test that allows the early detection of such resistance. An option to block this mechanism is to use drugs of different therapeutic classes for a single virus [49]. For example, HSV develops resistance against acyclovir, an efficient antiviral nucleoside analogue. HSV resistance can manifest as a mutation in the viral genes encoding thymidine kinase, which phosphorylates acyclovir to activate it. Without this phosphorylation, acyclovir has no therapeutic activity. However, these resistant viruses consequently become resistant to other drugs of the same therapeutic class, such as famciclovir, ganciclovir, and penciclovir, which have the same mechanism of action and require thymidine kinase for their activation [50].

Based on the mechanism of antiviral action, there are different aspects of developing drug resistance. Antiviral activity can target cellular or viral proteins, according to the nature of the virus, its mechanism of action, and the functioning of the host cells. Antiviral drugs are more specific when they target viral proteins. Such compounds are less toxic but have a narrow spectrum of antiviral activity, and the virus is more likely to develop drug resistance. In contrast, when the cellular proteins are targeted, the toxicity is higher. However, there is a wider activity spectrum and less chance for the drug to develop resistance [51].

### 2.3. Different Types of Antivirals

Antiviral drugs may have an inhibitory effect on the entire replication cycle. They can be classified according to the stage to which they are targeted: cell entry inhibitors [52,53,54,55,56,57], replicant inhibitors [40,58,59,60,61,62,63,64] and assembly, maturation, and release inhibitors [65,66,67,68,69]. Within a therapeutic class, antiviral agents can have different effects. List of commercialized antiviral compounds is presented on Table 1.

#### 2.3.1. Cell Entry Inhibitors

**Figure 1:**
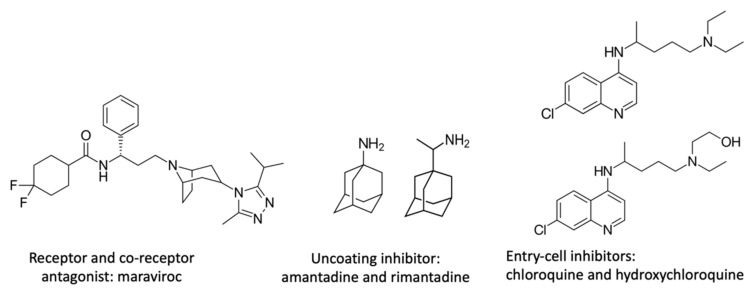
Chemical structure of different cell entry inhibitors.

To prevent virus replication, the first option is to avoid its entry into the host cell. There are different types of inhibitors. Maraviroc inhibits HIV entry by binding selectively and specifically to the CCR5 receptor on the CD4 cell surface [52]. Enfuvirtide is a synthetic peptide that blocks the HIV envelope from fusing with the CD4 cell membrane [53]. Other analogous antiviral agents such as amantadine and rimantadine (Figure 1), which have been used for several years against influenza A, bind to the protein ion channel (M2). The virus uncoating is no longer possible because the influenza virus uses this pH acidification channel to release its RNA fragments. Unfortunately, resistance has developed, and both drugs are currently ineffective [54,55].

Anionic compounds can also inhibit virus replication by avoiding virus adsorption (attachment) on the cell surface. For HIV, HSV, or another enveloped virus, anionic compounds can interact with the positively charged amino acid present in the viral surface glycoprotein, thus avoiding bonding with the host cell receptor. With this mechanism, viral entry by the fusion of the virion envelope with a cell membrane is not possible [30]. Anionic polysaccharides are a part of those types of inhibitors. This subject will therefore be further explored in the following section of this review paper.

#### 2.3.2. Replication Inhibitors

**Figure 2:**
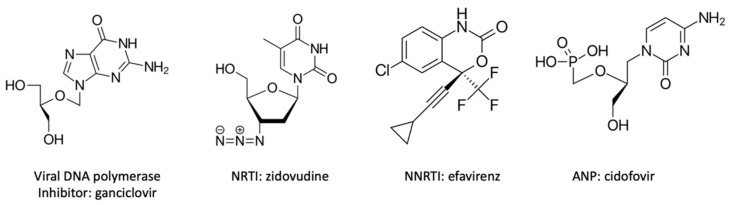
Chemical structures of different replication inhibitors. NRTI: nucleosides reverse transcriptase inhibitor; NNRTI: non-nucleoside reverse transcriptase inhibitor; ANP: acyclic nucleoside phosphonate.

Antiviral compounds can also affect replication enzymes. Most of these are nucleoside analogues and function by competitive inhibition. They are classified according to their target enzyme (Figure 2). Viral DNA polymerase RNA-dependent inhibitors are prodrugs. This means that analogue nucleoside drugs need to be phosphorylated to become active, once by virus kinase and twice by cellular kinase. Reverse transcriptase inhibitors (NRTIs: nucleosides reverse transcriptase inhibitors) have a similar mechanism of action, but they are antiretroviral and act against HIV virus. There are also non-nucleoside reverse transcriptase inhibitors (NNRTIs), which operate against HIV. Molecules, which are structurally different, bind noncompetitively on the reverse transcriptase to an active site, such as a hydrophobic pocket. The inhibition does not occur by competition with the natural deoxynucleotides triphosphates, but NNRTIs alter the mobility or conformation of the reverse transcriptase and the complexes become inactive [61]. The last category is acyclic nucleoside phosphonates (ANPs), which have a rather broad spectrum of activity, a longer action, and a lower chance of resistance. They are also active against many viruses. They only need two phosphorylations to be converted into their active form. ANP dephosphorylated analogues are also substrates for the DNA polymerase. The DNA elongation chain is stopped after the incorporation of a phosphorylated ANP, which acts like a chain terminator [71].

#### 2.3.3. Post-Replication Inhibitors

**Figure 3:**
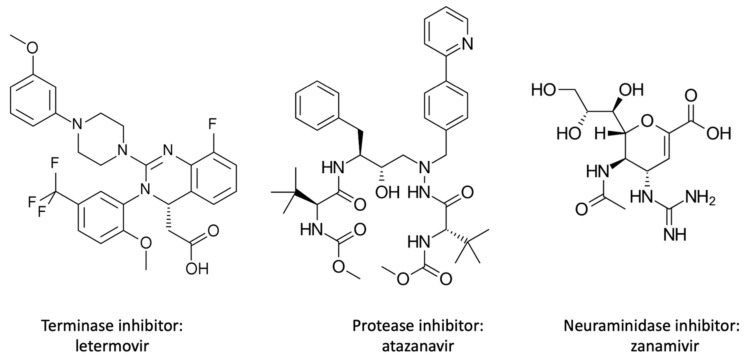
Chemical structure of different post-replication inhibitors.

The last stages that antivirals can target are those post-replication—namely, assembly, maturation, and release (Figure 3). Terminase inhibitors inactivate the human cytomegalovirus (HCMV) terminase enzyme complex, which catalyzes the cleavage and packaging of viral DNA. There are several maturation inhibitors that target the protease. Protease inhibitors prevent virus polypeptide cleavage by binding to the active site of the viral protease. This results in the formation of inactive and noninfectious virions. The last group of antivirals that needs to be discussed is the release inhibitors that target the viral neuraminidase. Neuraminidase releases the influenza virion by cleaving the bond between the virion and sialic acid present on host cell glycoproteins [68].

Different antiviral drugs can also be used in polytherapy. The utilization of different antivirals reduces the probability of resistance. Since the end of the 1990s, combined antiretroviral therapy has been recommended for the treatment of HIV-1. Non-nucleoside agents and protease inhibitors should be taken together due to the high probability of resistance. A nucleoside analogue reverse transcriptase inhibitor can also be added. This polytherapy is called HAART, high active antiretroviral therapy, and it is often a tri-therapy [72].

Only half of the antiviral drugs shown in Table 1 are still effective against viruses. That explains the urge for developing new antiviral compounds and the increasing interest in antiviral polysaccharides, as discussed in the following chapter.

## 3. Polysaccharides with Antiviral Activities

Polysaccharides represent a broad class of biological macromolecules with a range of diverse physicochemical properties. They are composed of monosaccharide units forming a polymeric carbohydrate through glycosidic linkages. They are essential for cell life activities because they play an important role in the maintenance of structure, energy storage, and cellular communication. Several studies have shown that natural and chemically modified polysaccharides can contribute to other types of biological activities, such as anticoagulant, antidiabetic, antioxidant, antitumor, immunomodulatory, anti-inflammatory, and especially antiviral activity [73]. Recently, the China Food and Drug Administration approved some patent medicines that contain polysaccharides in different pharmaceutical forms, such as capsules, tablets, oral liquids, and injections. Polysaccharides have been derived from *Coriolus versicolor*, *Poria cocos*, *Polyporus umbellatus*, *Lentinula edodes*, and *Lentinula edodes.* These drugs are involved in the treatment of chronic hepatitis and some immune disorders and are also used to reduce the side effects of chemotherapeutic agents [74].

Through the chemical modification of their structure, a huge variety of possible untapped structures can be created for the purpose of antiviral therapy. Their advantages are that they possess potent antiviral activity, low toxicity, and weak side effects and can be obtained from a wide range of sources.

### 3.1. Identification of the Main Polysaccharides with Antiviral Properties

Polysaccharides can be divided into different types according to their source: animal polysaccharides, algal and plant polysaccharides, mushrooms polysaccharides, microbial polysaccharides, and semisynthetic polysaccharide derivatives. The most studied antiviral polysaccharides are the marine ones. Marine organisms are rich in sulfated polysaccharides and have a wide range of pharmacological effects, including antiviral activity. The degree of sulfation of monosaccharides is important for the antiviral activity, and sulfation enhances the antiviral potency [75,76]. However, there is not sufficient evidence of the effectiveness of cellulose sulphate gel in preventing the vaginal transmission of HIV, gonorrhea, or chlamydial infection from man to woman, according to the results from a phase III trial in Nigeria [77]. The data available in both primary and secondary sources concerning the antiviral effects of natural polysaccharides are summarized in Table 2.

#### 3.1.1. Antiviral Activities of Polysaccharides That Have Animal Origins

**Figure 4:**
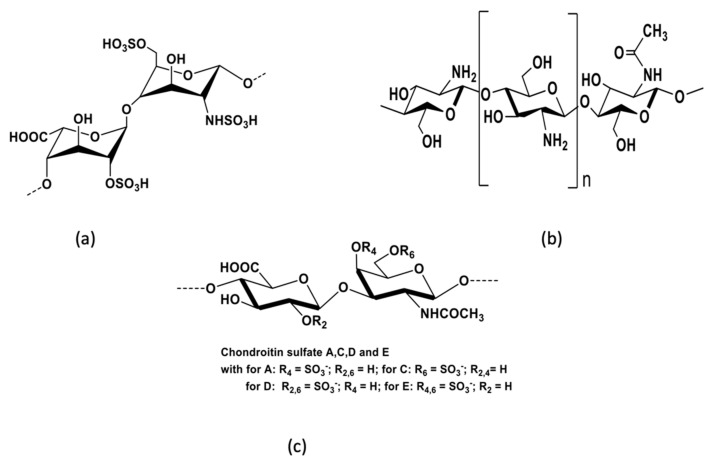
Main structures of antiviral polysaccharides isolated from animals: (**a**) heparin, (**b**) chitosan, and (**c**) chondroitin sulfate.

Animal polysaccharides are found in animal tissues and organs. The most studied antiviral animal polysaccharides are heparin, chondroitin sulfate, and chitosan. Heparin is a glycosaminoglycan that is usually derived from porcine intestine or bovine lung and consists of sulfated repeating disaccharide units. The most common disaccharide unit is composed of 2-O-sulfate-*α*-l-iduronic acid and 6-O-sulfate-*N*-sulfate-α-d-glucosamine linked by a 1,4-glycosidic bond (Figure 4). Heparin is widely used as an anticoagulant and antithrombotic agent. In addition, heparin also has antitumor, anti-inflammatory, tissue-protective, and antiviral effects [25,26,80]. In the 1990s, the potential of heparin to inhibit HIV was investigated following a report of HSV inhibition [126]. Neyts et al. have demonstrated that heparin can inhibit human cytomegalovirus by interacting with cell surface glycoproteins [79]. Recent studies have reported that heparin and its low-molecular-weight derivatives have an exceptional binding affinity to the spike protein of SARS-CoV-2 [78,80,127]. SARS-CoV-2 has an enveloped spherical shape. It is composed of a spike protein, envelope protein, nucleocapsid protein, and membrane protein. It is considered that the spike protein of SARS-CoV-2 can bind to ACE2 (angiotensin-converting enzyme 2) [27]. The prevention of the binding of the spike protein to ACE2 might play an essential role in future anti-SARS-CoV-2 strategies. For that reason, the inhibition of viral adhesion by heparin and low-molecular-weight heparins could provide important therapeutic opportunities.

Polysaccharides derived from marine fishes, shellfishes, and mollusks include chitosan and chondroitin sulfate. Chitosan is a linear polysaccharide that consists of randomly distributed d-glucosamine residues, acetylated or not, linked by *β*-1,4-linkages (Figure 4). It is obtained by the deacetylation of chitin found in crustacean shells. Chitosan is mainly used in biomaterials, pharmaceuticals, cosmetics, and agriculture [128,129,130]. Some studies have reported that chitosan can induce resistance to viral infections in plants, inhibit viral infections in animal cells, and prevent phage infection in microbial cultures [62]. Chitosan can provide two essential benefits for antiviral therapy: firstly, it has a direct antiviral effect on some viruses by inhibiting viral infection; secondly, it has an effect through inducing the antiviral immune response. It is considered that the main key points involved in augmenting the antiviral immune responses by chitosan are the stimulation of the immune cells (macrophages, etc.), the increase in the number of phagocytes, the encouragement of the secretion of nitric oxide in phagocyte, the promotion of the migration of neutrophils, and the increase of the levels of systemic (IgG) and mucosal (IgA) humoral responses.

The antiviral activity of chitosan has been shown to increase as its molecular weight decreases [85]. Although chitosan has an underscored antiviral potential, nowadays it is mainly used as a vehicle for nanoparticle drug delivery systems. Most studies that explore chitosan antiviral activity have been performed with plants, animals, or cell cultures. The antiviral activity of chitosan has not been investigated in randomized clinical trials.

Chondroitin sulfate (CS), which is usually isolated from animal cartilage, is composed of alternating glucuronic acid and *N*-acetylgalactosamine (Figure 4). Glucuronic acid can be O-sulfated at C2 or rarely at C3, and *N*-acetylgalactosamine can be sulfated at O4 and/or O6. Chondroitin sulfates are divided into several types based on the sulfation of the disaccharide units. Chondroitin sulfate E, which is commonly isolated from squid cartilage, is predominantly 4-O- and 6-O-sulfated. Like heparin, chondroitin sulfate has long been recognized as an anti-HIV agent. Chondroitin sulfate can have diverse antiviral activities—for example, chondroitin sulfate isolated from sea cucumber inhibits HIV replication by interfering with the virus entry [131]. Furthermore, CS-E isolated from squid has been reported to have antiviral activity against HSV and DENV [83]. Atsushi Jinno-Oue and colleagues reported the significant potential of CS in the treatment of human T-cell leukemia virus type 1 (HTLV-1) in an in vitro study. According to the findings of this study, novel E type CS-like molecules would have great potential in the treatment of HTLV-1 [81].

In 2020, Shuang Song et al. reported that they had investigated the inhibitory activity against SARS-CoV-2 of CS in sharks, sea cucumber sulfated polysaccharide, fucoidan from brown algae, and iota-carrageenan from red algae. CS showed no anti-SARS-CoV-2 potential, while the sea cucumber sulfated polysaccharide, fucoidan, and carrageenan showed significant anti-SARS-CoV-2 activity at concentrations of 3.90–500 μg mL^−1^. The study was performed on cell culture [82]. A variety of novel marine polysaccharide structures with antiviral effects have been obtained from marine shellfishes and are expected to be new candidates for antiviral drugs [75]. The structure of shellfish polysaccharides is very complex because of the diversity of linkage types and the variety of compositions of monosaccharides. Shellfish polysaccharides usually include many sulfates.

Chanathip Thammakarn et al. performed an in vitro study that investigated the efficacy of scallop shell powders for inactivating avian influenza virus (AIV). The researchers reported that scallop shell powder was able to inactivate AIV during a short contact period (3 min), even under harsh conditions [132].

Woo et al. isolated polysaccharides from Korean edible clams with virus–cell fusion inhibitory activity against HIV infection [88]. In 2021, Fei Tang et al. reported significant anti-hepatitis B virus activity with low toxicity, associated with the polysaccharides from *Thais clavigera* [133].

#### 3.1.2. Antiviral Activities of Polysaccharides from Seaweeds, Mushrooms, and Plants

Polysaccharides are usually found in the cell wall of plants, seaweeds, and mushrooms and are composed of monosaccharides with *α*- or *β*-glycosidic linkages. Seaweed polysaccharides have received attention due to their biological properties, such as antioxidative, anti-inflammatory, anticancer, and antiviral effects [134], as well as their availability in nature. They represent a rich resource of antiviral polysaccharides, including carrageenans from red algae, ulvans from green algae, and alginates, fucans/fucoidans, and laminarins from brown algae. Seaweeds are widely available in nature and constitute an abundant resource of antiviral polysaccharides. Carrageenan isolated from *Meristiella gelidium* was found to have potent inhibitory effects in vivo against HSV and murine cytomegalovirus [115].

**Figure 5:**
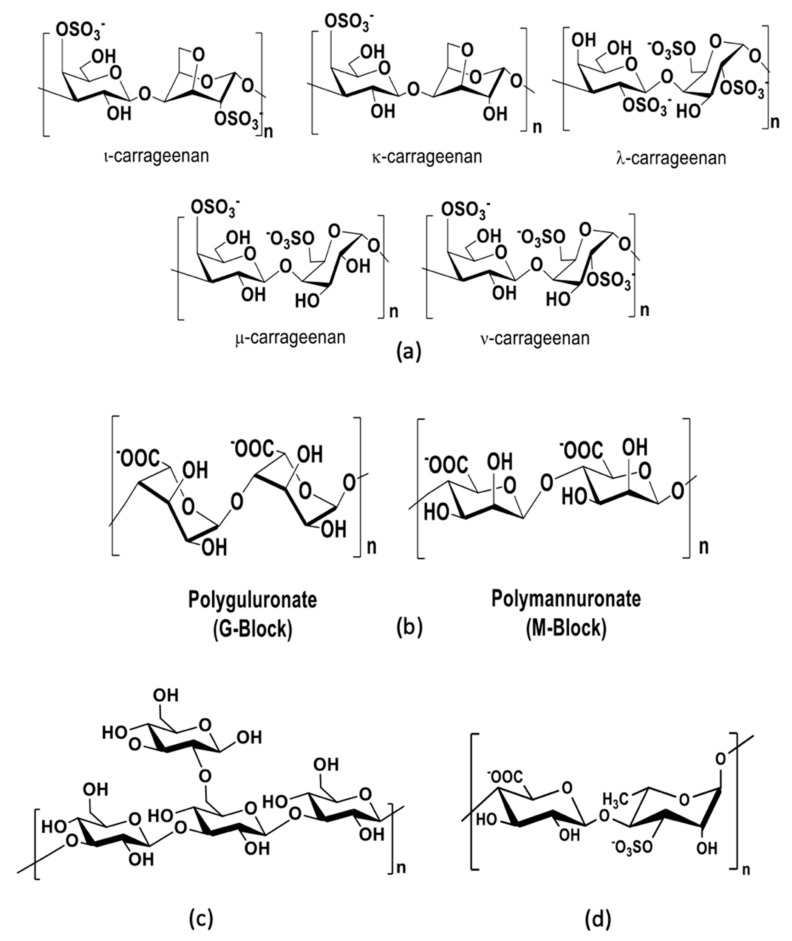
Main antiviral polysaccharides isolated from algae: (**a**) carrageenans, (**b**) alginates, (**c**) laminarin, and (**d**) ulvans.

Carrageenans are extracted from the cell wall of red edible seaweeds and are widely used in the food and cosmetic industries as thickeners. They are composed of repeating sulfated d-galactose units and 3,6-anhydrogalactose units, both sulfated and unsulfated. Carrageenans are classified into six basic forms—lambda (*λ*), kappa (*κ*), iota (*ι*), mu (*m*), nu (*n*), and theta (*q*)—according to the monosaccharide composition and the position of sulfate groups (Figure 5). The natural forms of carrageenans found in red algae are the lambda (*λ*), kappa (*κ*), and iota (*ι*) forms. Numerous studies have shown that the antiviral activity of *ι*-carrageenan is more effective than that of *λ*- and *κ*-carrageenans [97,102,103]. The antiviral effects of carrageenans are closely related to their molecular weights and degree of sulfation [75]. Carrageenans have antiviral effects on several enveloped and nonenveloped viruses by inhibiting the internalization or binding of the virus in the cells [95,97,103]. A new family of carrageenans with antiviral properties has been reported, consisting of beta-carrageenan, which is analogous to kappa-carrageenan but without the sulfate on the C4 of the 1,3 units. Beta-carrageenan can induce the suppression of viral infection in tobacco leaves infected with tobacco mosaic virus [103].

Alginates are linear polysaccharides found in the cell wall of brown algae and are commonly used in the manufacture of paper and textiles. Alginates have also found numerous applications in cosmetics, the food industry, and biomedical science and have been particularly attractive due to their antiviral activities. They are composed of a central backbone of poly-d-guluronic acid (G blocks), poly-d-mannuronic acid (M blocks), and alternating residues of d-guluronic acid and d-mannuronic acid (GM blocks) [135] (Figure 5). Alginates have shown a high antiviral effect against tobacco mosaic virus infection, and their inhibitory effect increases at lower mannuronate-to-guluronate ratios [93]. A prominent alginate drug from marine seaweeds named 911 has shown promising antiviral activity against HIV. The polysaccharide 911 is derived from alginate with an M:G ratio of 4:1. Xin et al. reported that 911 inhibited the reverse transcriptase and interfered with the adsorption of the virus to cells [136].

Laminarin, also known as laminaran, is a storage polysaccharide found in brown seaweeds. It is composed of repeating 20–25 glucose units linked by *β*-(1,3)-glycosidic linkages with *β*-(1,6)-branches (Figure 5). Laminarin is created by photosynthesis and has a good inhibitory effect on virus proliferation with a low toxicity [75]. Muto et al. reported that laminarin extracted from kelp inhibited the adsorption of HIV on lymphocytes and inhibited the activity of HIV reverse transcriptase [137].

Fucans, a major constituent of brown seaweeds, are sulfated polysaccharides with a high molecular weight. Fucans are composed of a central backbone of l-fucose composed of (1,3)-*α*-l-Fuc or alternating (1,3)-*α*-l-Fuc and (1,4)-*α*-l-Fuc; sometimes, (1,2)-*α*-l-Fuc is present. Sulfate groups are often present in positions C2 and/or C4. Several studies on the structural composition of fucans from brown algae have shown that fucans are very complex, and their structures vary from species to species [138]. Neutral sugars such as galactose, glucose, mannose, and uronic acid can occur in the central backbone. Fucans are classified into three major groups—fucoidans, xylofucoglycuronans, and glycuronogalactofucans—according to their central backbone, branch composition, linkage mode, sulfate content, and position. Fucoidans have shown relevant biological activities, such as anticoagulant, antioxidant, antithrombotic, antitumor, and antiviral effects. Queiroz et al. showed that fucoidan extracted from *Dictyota mertensii*, *Lobophora variegata*, *Fucus vesiculosus*, and *Spatoglossum schroederi* could inhibit HIV reverse transcriptase activity [89].

In vivo studies have shown that fucoidans (native fucoidan isolated from *F. evanescens* and its derivative obtained after enzymatic modification) protect against intravaginal HSV-2 infection in mice [139]. Ulvans are the major water-soluble polysaccharides found in the cell wall of green seaweeds [105,106,108]. They are highly sulfated and are mainly composed of a repeating disaccharide unit through an l-rhamnose 3-sulfate linked to d-xylose, d-xylose 4-sulfate, d-glucuronic acid, and l-iduronic acid (Figure 5). Ulvans display several biological activities, such as antioxidant, antitumor, and anticoagulant activity. They are also of potential interest due to their food, pharmaceutical, agricultural, and chemical applications [140]. Aguilar-Briseño et al. reported that ulvans extracted from *Ulva clathrata* had antiviral activity against Newcastle disease virus in vitro by the inhibition of viral fusion.

Polysaccharides extracted from microalgae include naviculan and some sulfated polysaccharides. Naviculan is a sulfated polysaccharide isolated from green microalgae (diatom) composed of several sugars such as mannose, galactose, rhamnose, xylose, fucose, and sulfate groups. Lee et al. demonstrated the potent antiviral activity of naviculan against HIV, HSV-1, HSV-2, and IFV by inhibiting viral replication [109]. The microalga *Gyrodinium impudicum* produces a highly sulfated polysaccharide, pKG03, composed of galactose linked with uronic acid and sulfate groups. pKG03 can induce the inhibition of viral adsorption and internalization for IAV and EMCV [109]. Polysaccharides A1 and A2 are found in the marine microalga *Cochlodinium polykrikoides*. They have been shown to contain galactose, glucose, mannose, uronic acid, and sulfate groups. Their antiviral activity has been demonstrated against enveloped viruses, such as HIV-1, HSV-1, IFV-A, IFV-B, RSV-A, RSV-B, and PIFV-2 [111].

Polysaccharides extracted from edible, medicinal plants and mushrooms are structurally diverse and have a heterogenous monosaccharide composition. They are mainly composed of glucose (Glc), mannose (Man), arabinose (Ara), talose (Tal), xylose (Xyl), ribose (Rib), rhamnose (Rha), sorbose (Sor), fructose (Fru), fucose (Fuc), galactose (Gal), glucuronic acid (GlcA), and galacturonic acid (GalA) [141]. They can inhibit viruses by interfering in several steps of the virus life cycle. Polysaccharides isolated from mushrooms have shown potential antiviral activity—for example, against HIV—by the inhibition of the reverse transcriptase and proteases [118]. In Table 3, data obtained from recent studies (2020–2021) on the antiviral activity of some polysaccharides derived from seaweeds, plants, and mushrooms are summarized. The sulfated polysaccharide lambda-carrageenan, obtained from marine red algae, demonstrated promising antiviral activity against influenza viruses and severe acute respiratory syndrome coronavirus [97,142]. This compound has great potential to become a novel antiviral agent for the treatment of infections caused by several respiratory viruses. Astragalus polysaccharides (APS), derived from *Astragalus membranaceus*, could be used as an immunomodulator to enhance immune responses. In 2021, Yumei Zhou et al. reported that they had analyzed the effects of APS on the immune response to ovalbumin in mice. APS has been shown to enhance Th1 and Th2 immune responses to OVA in BALB/c mice. It has been suggested that APS is a nontoxic, promising candidate for use as a vaccine adjuvant [143].

#### 3.1.3. Antiviral Activities of Microbial Polysaccharides

Microbial polysaccharides are produced in the metabolic process by microorganisms such as cyanobacteria, bacteria, and fungi. Microbial polysaccharides are a promising new source of bioactive products.

The extracellular polysaccharides (EPS) synthetized by microorganisms are structurally and functionally diverse. They mostly provide protection against negative environmental conditions. These polysaccharides are produced in a short amount of time and their extraction is quite easy, which enables their use in various fields. They have various applications in the food, cosmetics, and textile industries. Moreover, they have various biologicals effects, such as antioxidant, antimicrobial, and antiviral activities [141]. Arena et al. isolated an EPS with anti-HSV-2 activity from the bacteria *Geobacillus thermodenitrificans* from a shallow marine vent of Vulcano Island [149]. An EPS isolated from *Paecilomyces lilacinus* was shown to inhibit the adsorption and biosynthesis of HSV-1 in Vero cells.

Calcium spirulan is a sulfated polysaccharide isolated from the cyanobacteria *Spirulina platensis*, which is composed of two types of disaccharide repeating units, O-rhamnosyl-3-O-methylrhamnose and O-hexuronosyl-rhamnose. Glucuronic acid and galacturonic acid were also found in calcium spirulan [150]. Mader et al. demonstrated that calcium spirulan inhibited HSV-1 infection in vitro by blocking viral attachment and penetration into host cells [122]. The spirulan-like molecules isolated from *Arthrospira platensis* showed antiviral activity against HCMV, HSV-1, HPV-6, and HIV-1 [151].

Nostoflan is also a polysaccharide isolated from a cyanobacteria, *Nostoc flagelliforme*. It is an acidic polysaccharide [125]. Nostoflan has been shown to have an inhibitory effect on the viral binding process against HSV-1, HSV-2, IAV, and HCMV [124,152].

#### 3.1.4. Antiviral Activities of Polysaccharide Derivatives

The biological activity of polysaccharides is related to their structure. Numerous studies have shown that after specific chemical modifications, polysaccharides can have a stronger effect or show new biological activities. Antiviral studies of polysaccharide derivatives provide a broad prospect for antiviral drug development. The main chemical modification methods used include sulfation, phosphorylation, complexation, and enzymatic modification. The polysaccharide derivatives with antiviral effects are summarized in Table 4.

Sulfated polysaccharides are the most studied class of antiviral polysaccharides. Polysaccharides that are naturally sulfated, such as heparin, chondroitin sulfate, carrageenan, and ulvans, have strong antiviral activity. Polysaccharides chemically modified with sulfate or sulfonate groups have a strong activity concerning blocking herpes simplex virus infections [129]. Some natural polysaccharides have shown good antiviral activity after sulfation. For example, dextran sulfate, a polysaccharide chemically modified by sulfation, could inhibit the replication of enveloped virus and the fusion of influenza virus with cell membranes [153,154,155,173]. The dextran polysaccharide is produced by several bacteria, but only *Leuconostoc dextranicum* and *Leuconostoc mesenteroides* are used for commercial purposes. Dextran is composed of a backbone of *α*-(1,6)-linked d-glucose unit with the *α*-(1,3) and *α*-(1,4) branches of glucose. However, the pharmacological properties of polyanions (including sulphated polysaccharides) in vivo are limited due to the low bioavailability of the drugs to their viral targets and therefore poor antiviral activity. Their incorporation into drug delivery systems would enhance their therapeutic benefits as antiviral agents [56]. Moreover, the chemical modification of chitosan and chitin can generate compounds with good pharmacological activities, such as antiviral effects. For example, Sosa et al. synthetized a sulfated polysaccharide derivative of chitin, *N*-carboxymethylchitosan-*N*-O-sulfate (NCMCS). NCMCS has an inhibition action against HIV-1 and RLV [164]. Recently, a new amino-derivatized chitosan that showed good biological activities, such as antioxidant, antimicrobial, and antiviral effects, was developed [174]. Among amino-derivatized chitosans, aminoethyl-chitosan showed anti-HIV activity and constituted a new-generation drug candidate against HIV [172]. Chitosan can also be hydrolyzed through an enzymatic reaction to obtain chitooligosaccharides. These have been reported to have a good water solubility, high absorption profile, and various biological activities [175]. Sulfated chitooligosaccharides, synthetized by a random sulfation modification, have been reported to have a good anti-HIV activity [172].

Phosphorylated polysaccharides are limited in nature, so synthetic methods are used in their production. Phosphorylated polysaccharides have attracted attention because of their antiviral properties. Some studies have demonstrated that the phosphorylated forms have a stronger inhibitory effect against duck hepatitis A virus (DHAV) compared to the natural forms [167,168]. Feng et al. found antiviral activity against CPV in a chemically phosphorylated polysaccharide isolated from *Cyathulae radix* [168].

### 3.2. Structure–Activity Relationships

The antiviral activity of polysaccharides can be explained by a combination of structural factors. These factors depend on the carbohydrate backbone, including the molecular weight, linearity, flexibility, and hydrophobic sites, and on the anionic groups, including the degree of sulfation and the distribution of sulfate groups in the backbone. The most explained factor in the literature is the polyanionic nature of polysaccharides, as well as the type of anionic groups present on the polysaccharide. Research over the last 20 years has shown that sulfate groups are in most cases required for the antiviral activity and the degree of sulfation has a major impact on it.

Natural sulfated polysaccharides are known to inhibit enveloped viruses. The inhibitory effect of sulfated polysaccharides increases with the molecular weight, and the highest activity is generally in the range of 10–100 kDa [176]. For example, the chondroitin sulfate E (~70 kDa) is much larger than heparin (~12.5 kDa), and the anti HSV-1 effect of chondroitin sulfate E (IC_50_ = 0.06 to 0.2 μg mL^−1^) is stronger than that of heparin (IC_50_ = 1.0–0.8 μg mL^−1^) [83]. However, Liu et al. showed that the sulfated polymannuroguluronate (SPMG), a low-molecular-weight (~10 kDa) sulfated polysaccharide, can have a strong antiviral activity with three sulfates per disaccharide unit [177]. Therefore, low-molecular-weight sulfated polysaccharides with a high sulfate content can have strong antiviral activities, which demonstrates that the molecular weight is not an absolute factor.

The degree of sulfation (i.e., the number of sulfate groups per monosaccharide residue) is an important parameter for the antiviral activity. For natural polysaccharides, the antiviral effect increases as the degree of sulfation increases. For example, sulfated seaweed polysaccharides with a degree of sulfation lower than 20–22% do not show an antiviral activity [176]. Moreover, the antiviral activity of *κ*-carrageenan is more effective than that of other types of carrageenans, and this can be explained by the degree of sulfation [152]. The distribution of sulfate groups is also important. For example, the chondroitin sulfate E, a sulphated polysaccharide at position 4 and 6, exhibited antiherpetic activities, and data have shown that chondroitin sulfate with sulfate groups at other positions (type A, B, C, and D) exhibited either little or no anti-HSV activity [83]. The specific positioning of the sulfate groups of chondroitin sulfate E can explain this antiherpetic activity.

Other anionic groups can be present on polysaccharides and can confer antiviral activity. The presence of alkyl groups increases the antiviral activity through the formation of hydrophilic–hydrophobic structures [176]. However, uronic acid residues and carboxyl groups have shown very little antiviral activity [178]. Recent studies have shown that the nature of the counter cations of anionic sites plays an important role in the antiviral activity [153]. The replacement of Na^+^ by another metal cation decreased the antiviral activity of calcium spirulan against HSV. Therefore, the type of anionic group is important, and the polyanionic nature of polysaccharide is a main factor in antiviral activity. Sulfate groups are, in many cases, required for the antiviral activity.

Heparinoids are sulfated polysaccharides similar to heparin in structure and generally include ulvans, chondroitin sulfate, carrageenans, fucoidans, and sulfated derivatives such as dextran sulfate. Heparin, with four negative charges for each disaccharide unit, has the highest negative charge density from the natural polymers. Heparinoid polysaccharides can interact with a range of proteins according to their negative charge obtained through sulfate groups. They can interact with the positive charge region of glycoproteins on the surface of cells, which leads to a shielding effect and prevents the binding of viruses to the cell surface [79]. For example, heparan sulfate is a glycoprotein required for infection by human papillomavirus [179]. Capsid proteins from human papillomavirus interact with heparin and cell surface glycoproteins. The interaction between human papillomavirus and the cell surface can be inhibited by heparin and carrageenans [95]. By employing selectively desulfated heparin, it was shown that each sulfate group was important for viral binding [180]. Moreover, Copeland et al. synthetized a 3-O-sulfated heparin with an anti-HSV-1 activity that demonstrated that heparin required a unique sulfation moiety to inhibit HSV-1 [156].

Carrageenans are polysaccharides with a high molecular weight and poor tissue penetration that have limited potential antiviral application in humans. Their antiviral activity is very broad, they can inhibit enveloped and nonenveloped viruses, and the inhibitory action is usually different according to the different types of carrageenans. O-acylated carrageenans with different molecular weights have a stronger inhibitory effect against HIV-1 by depolymerization and sulfation [172]. Moreover, carrageenan oligosaccharides obtained by enzymatic degradation show increased bioactivity and bioavailability when a smaller molecular weight is achieved [180]. Wang et al. reported that low-molecular-weight carrageenan oligosaccharides could inhibit IAV, and a structure–activity relationship study showed that the sugar length, specific sugar linkage, and sulfate content might be the main factors of *κ*-carrageenans’ anti-IAV activity [165]. The highest antiviral activity of *κ*-carrageenans is in the range of 1–3 kDa with a sulfate content of 0.8–1.0 mole/mole of disaccharide [165].

Dextran sulfate mainly inhibits enveloped viruses, and low-molecular-weight dextran sulfate is inactive against IAV, HSV, and some other viruses. The inhibitory effect of dextran sulfate with a molecular weight of 1 kDa against HIV depends on the cell type and the virus strain [181]. The antiviral activity of fucans could be due to the hydrophobic character obtained by methyl groups [176]. The sulfation of fucans is also necessary for their activity. Carboxyl reduction in the glucuronic acids to glucose units in fucans suppresses their antiviral activities. Ivanova et al. noted that ulvans had a good antiviral effect against IAV and demonstrated that this effect is dose-dependent, strain-specific, and selective [182]. Scallop skirt glycosaminoglycan isolated by Yu et al. showed an anti-HSV-1 activity at different concentrations, and the antiviral activity increased with the duration of action [183].

It has been shown that the antiviral activity of chitosan against plant viruses varies according to the chitosan source and the plant species [184]. Its antiviral activity increases as its molecular weight decreases, and the highest antiviral activity of chitosan is in the range of 1.2–2.2 kDa [85]. Small chitosan molecules can possibly have a better penetrating ability in plants. Moreover, the antiviral activity of chitosan against bacteriophages increases as the molecular weight increases, and chitosan with a molecular weight over 200 kDa is able completely to reduce titers of bacteriophages. Furthermore, the antiviral activity of low-molecular-weight chitosans produced by enzymatic degradation significantly increases with the lowering of their polymerization degree [185]. The chemical modification of sulfated chitin showed that its inhibitory effect against HIV-1 depends on the sites of sulfation [86,128].

### 3.3. The Mechanisms of Antiviral Polysaccharides

**Figure 6:**
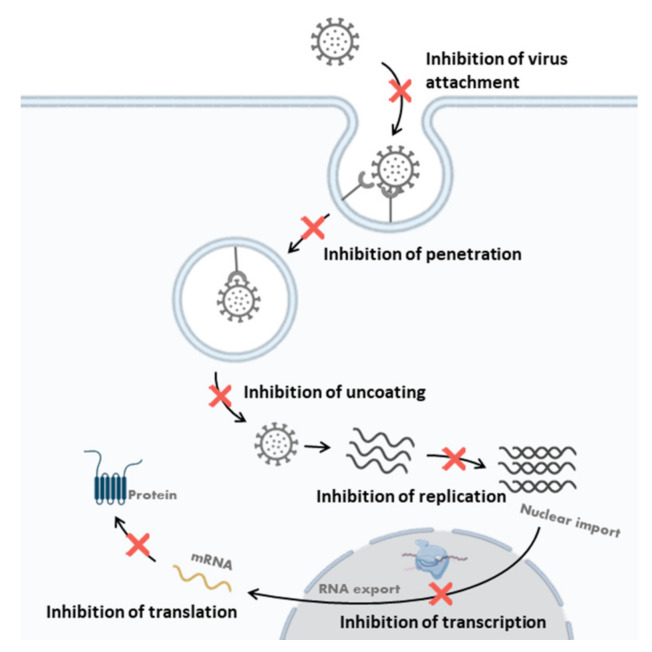
Steps of viral replication inhibition by antiviral polysaccharides.

Polysaccharides can either inhibit viruses through a direct antiviral effect, interfering with the viral cycle, or improve the host antiviral immune responses. In this paper, the direct antiviral activities of polysaccharides are described. Polysaccharides can directly inhibit a virus before infection and inhibit the viral life cycle at different stages. The antiviral mechanism of action is related to the structure of the polysaccharides. The steps of viral replication inhibition by antiviral polysaccharides are proposed in Figure 6 and summarized in Table 2 and Table 4.

#### 3.3.1. Killing the Virus Directly

Some polysaccharides can enter the cells and have a direct virucidal activity. Carrageenan might inhibit enveloped virus by direct action on the virus surface through its negative charge. Carlucci et al. found that *λ*-type carrageenan could bind to HSV, resulting in the inactivation of HSV replication [186]. The direct virucidal effect of carrageenan polysaccharides could be due to the irreversible binding of carrageenan with virions, taking the sites on viruses required for viral attachment to host cells [176].

Moreover, chitosan polysaccharides and oligosaccharides were reported to show the direct inactivation of two human enteric viral surrogates: FCV-F9 and bacteriophage MS2. Additionally, the antiviral effect of MS2 is molecular weight-dependent [186].

#### 3.3.2. Inhibition of Virus Attachment

The first step of virus invasion is binding to the host cell surface through electrostatic interactions. Based on recent data, sulfate could be successfully used in the prevention of virus entry. As virus entry into the host cells is the key point of every viral infection, the inhibition of such a mechanism would be of great importance for the current antiviral strategies [127,187]. One of the key points of the inhibition of viral attachment is the anionic nature of polysaccharides. Sulfated polysaccharides can interfere with the viral adsorption process by blocking positive charges on the cell surface.

#### 3.3.3. Inhibition of Penetration and Uncoating

The internalization process usually involves endocytosis, the fusion of virus with the cell membrane, and the translocation of the virus. For most cells, the uncoating step occurs after viral internalization and, for some phages, the uncoating step occurs at the same time as internalization. Some polysaccharides, especially sulfated polysaccharides, can block the virus internalization and uncoating by interfering with the allosteric process of the virus particles.

Carrageenans with an anti-HPV effect can directly bind to the HPV capsid to inhibit the penetration and uncoating processes [95]. Moreover, the release of DENV from endosomes after entering the host cell may be interfered with by *λ*- and *ι*-carrageenans. The inhibition of the uncoating process by *ι*-carrageenans may be due to the direct interaction of the polysaccharides with the virus membrane glycoprotein E [96,97]. Grassauer et al. reported that the mechanism of action of *ι*-carrageenan against HRV could be due to the interference of the allosteric process during HRV penetration [188].

Mechanism studies of the polysaccharide p-KG03 isolated from *Gyrodinium impudium* suggest that p-KG03 can inhibit the binding of IAV to host cells, prevent the internalization step, and block early viral replication [110]. Moreover, naviculan was demonstrated to inhibit HIV, HSV-1, HSV-2, and IFV by interfering with the initial stages of viral adsorption and internalization [109]. Calcium spirulan was found to inhibit the penetration of various enveloped viruses, including HSV-1, HCMV, measles virus, mumps virus, IAV, and HIV-1 [122,123,150].

#### 3.3.4. Inhibition of Viral Transcription and Replication

Virus transcription and replication can be inhibited through interference with viral replication enzymes or other intracellular targets.

Polysaccharides are able to interfere with viral replication-related enzymes and relevant targets in host cells. For example, it was reported that iota-carrageenan inhibits porcine reproductive and respiratory syndrome virus replication at both the mRNA and protein levels [189,190].

Gonzalez et al. analyzed the mechanism of action of carrageenan against HSV-1 and found that carrageenans could inhibit a step in virus replication [191]. In addition, *ι*-carrageenan appeared to inhibit the replication of DENV in mosquito cells [192]. Wang et al. demonstrated that low-molecular-weight *κ*-carrageenan oligosaccharides could inhibit the replication of the influenza A H1N1 virus in vivo and in vitro [165,193]. They demonstrated that the carrageenan oligosaccharide CO-1 could inhibit IAV mRNA transcription and protein translation [193].

In addition, some polysaccharides from brown algae, such as fucoidan, alginate, and laminarin, could inhibit HIV reverse transcriptase, leading to an antiviral effect. Queiroz et al., in particular, demonstrated a pronounced avian reverse transcriptase inhibitory effect in vitro on 0.5–1.0 μg/mL fucoidan isolated from *F. vesiculosus* and an inhibition of the reverse transcriptase activity (51.1%) on activated DNA using alginic acid [89]. Moreover, in their study, Muto et al. showed that laminarin extract could also be used to inhibit HIV reverse transcriptase [137]. Finally, it should be mentioned that alginate derivatives have been described as potential inhibitors of the transcription of HIV-1 in a dose-dependent manner and have been shown to block the binding of HIV-1 to MT (4) cells [136].

## 4. Conclusions and Future Prospect(s)

Viruses are the cause of several diseases that can cause serious conditions in certain cases. The HIV and Ebola viruses and, more recently, SARS-CoV-2 are examples of highly pathogenic viruses that can lead to death. Vaccines are a possible solution against viruses, and thanks to these, some virus diseases have been eradicated, as in the case of smallpox. However, in some cases, they are not sufficiently effective, or their development takes too long a time. Therefore, it is essential to have effective solutions to slow down the spread of viral infections. Humanity still needs novel, specific, and clinically effective drugs for many viral infections, including SARS-CoV-2. It is important that these molecules show not only a high efficacy against viruses, but also a low toxicity for human cells.

Polysaccharides are new natural agents that could potentially be used in the treatment of virus infections due to their medicinal properties. The major advantages of polysaccharides are their high efficacy, high biocompatibility, and low toxicity. They are widely available in nature, so their production is largely low-cost. More research is needed to optimize the clinical application of polysaccharides against viruses due to their complex physical nature, which makes it difficult to understand their diverse mechanisms of inhibition in vivo. The chemical modification of the structure of polysaccharides could potentially improve their physiological activity and provides great promise. In the next generation of research on this topic, many scientists will need to continue to explore the potential of polysaccharides and their derivatives to discover new efficient antiviral biomolecules. However, most of the studies that have investigated polysaccharides have been performed in vitro. Therefore, the biological activity of these substances should be studied in more detail in randomized clinical trials.

## Figures and Tables

**Table 1:** Commercialized antiviral compounds.

Therapeutic Class	Mechanism	Type	Target Viruses	Antivirals	Reference
Cell entry inhibitors	Fusion inhibitors	Polyanions, nonspecific inhibitors	Enveloped viruses: HIV, HSV, CMV, RSV	Polyanions in development: cosalane derivatives, polyoxometalates, polycarboxylates polysulfates, polysulfonates, negatively charged albumins, sulphated polysaccharides	[56]
		Receptor and co-receptor antagonist analogues	HIV-1	Maraviroc	[52,54]
	Fusion and penetration inhibitors	Fusion inhibitor	HIV-1	Enfuvirtide	[53,55]
		Uncoating inhibitor	Influenza A	Amantadine, rimantadine	[57]
Replication inhibitors	Nucleosides analogue inhibitors (activation by triphosphorylation) with competitive inhibition	Viral DNA polymerase inhibitors	Herpes viruses (HSV-1, HSV-2, VZV, CMV, EBV, HHV-6, HBV)	Foscarnet, acyclovir, famciclovir, brivudine, valganciclovir, penciclovir, ganciclovir, entecavir, telbuvine	[40,58,59]
		Reverse transcriptase inhibitors	HIV	NRTIs: zidovudine, didanosine, zalcitabine, stavudine, abacavir, emtricitabine, lamivudine	[60,61]
	Non-nucleoside inhibitors			NNRTIs: efavirenz, nevirapine, delavirdine, etravirine	[62,63,64]
	Acyclic nucleoside phosphonates (activation by diphosphorylation)	Acyclic nucleoside phosphonates (ANP)	Herpes viruses (HSV-1, HSV-2, VZV, CMV, HHV-6, EBV, HBV), HIV-1, HIV-2	Cidofovir, adefovir, tenofovir	[70]
Assembly, maturation, and release inhibitors	Packaging inhibitor	Viral terminase enzyme complex inhibitors	CMV	Letermovir	[65]
	Maturation inhibitor	Viral protease inhibitors (polyprotein precursors cleavage)	HIV-1, HIV-2	Amprenavir, fosamprenavir, atazanavir, indinavir, lopinavir, nelfinavir, ritonavir, saquinavir, darunavir, tipranavir, atazanavir	[66,67]
	Release inhibitors	Viral neuraminidase inhibitors	Influenza A and B	Zanamivir, oseltamivir, pramivir	[68,69]

HIV: human immunodeficiency virus; HSV: herpes simplex virus; CMV: cytomegalovirus; VZV: varicella-zoster virus; RSV: respiratory syncytial virus; EBV: Epstein—Barr virus; HBV: hepatitis B virus.

**Table 2:** Polysaccharides from natural sources reported to have antiviral activities.

Origin	Polysaccharides	Antiviral Effects	Mechanisms	References
I. Animal				
Bovine and porcine tissues	Heparin	Anti-CMV, HSV, HPV, DENV, JEV, YFV, ZIKV, HIV	Inhibition of adsorption	[78,79,80]
Cartilage of animals	Chondroitin sulfate	Anti-HIV, HSV, HTLV-1, DENV	Inhibition of adsorption	[81,82,83,84]
Crustacean shell	Chitosan	Anti-bacteriophages, plant viruses, HIV, FCV-F9	Direct virucidal effect	[85,86]
Shellfish	Shellfish polysaccharide	Anti-HBV, HIV-1, IFV, HSV-1	Inhibition of adsorption	[87,88]
II. Algal and plant				
Brown algae	Fucoidan	Anti-HIV, DENV, IAV, NDV, HSV	Inhibition of adsorption and transcription	[89,90,91]
	Laminarin	Anti-HIV	Inhibition of adsorption and transcription	[92]
	Alginate	Anti-HIV, HBV, IAV, TMV	Inhibition of transcription	[93,94]
Red algae	*ι*-carrageenan	Anti-DENV, HAV, HRV, IAV, HPV, ECMV, HSV, SFV, ASV, VV	Inhibition of adsorption, penetration, uncoating, replication and transcription	[82,92,95,96,97,98,99]
	*κ*-carrageenan	Anti-enterovirus, IAV, HSV, CMV, Sindbis virus, VV, HIV, DENV, TMV	Inhibition of replication and transcription	[97,100]
	*λ*-carrageenan	Anti-DENV, HSV, HPV, HAV, CMV, HIV, Sindbis virus, VV, BoHV-1, SuHV-1, RABV	Direct virucidal effect, inhibition of adsorption	[95,97,101,102]
	*β*-carrageenan	TMV	-	[103]
Green algae	Ulvan	Anti-IFV-A, JEV, NDV	Inhibition of penetration and uncoating	[104,105,106,107,108]
Diatom (microalga)	Naviculan	Anti-HIV, HSV-1, HSV-2, IFV	Inhibition of adsorption	[109]
Microalgae	Sulfated polysaccharide	Anti IAV, HIV-1, HSV-1, IFV-A, IFV-B, RSV-A, RSV-B, PIFV-2, EMCV	Inhibition of penetration and replication	[76,78,110,111,112,113]
Edible	Edible polysaccharide	Anti-DEV, HSV-1	Inhibition of adsorption, penetration and uncoating	[114,115]
Medicinal plants	Medicinal plant polysaccharide	Anti-HSV-2, PV-1, PV-2, DHAV, BoHV-1, HBV, H1N1, H3N2, HIV, rotavirus	Inhibition of adsorption and replication	[116,117]
III. Mushroom				
Mushrooms	Mushroom polysaccharide	Anti-IFV-A *, PV-1, HIV-1, HBV, IHNV	Inhibition of transcription and replication	[118,119,120]
IV. Microbial				
Marine bacterias	EPS	Anti-HSV-2, HSV-1	Inhibition of adsorption	[121]
Cyanobacterias	Calcium spirulan	Anti-HSV-1, HCMV, measles virus, mumps virus, IAV, HIV-1	Inhibition of adsorption and replication	[122,123]
	Nostoflan	Anti-HSV-1, HSV-2, IAV, HCMV	Inhibition of adsorption	[124,125]

* Inhibitory effect observed in vivo.

**Table 3:** Studies investigating the antiviral activity of polysaccharides, 2020–2021.

Source	Type of Extract	Virus Involved	Object of Study	Antiviral Activity	Results	Reference
*S. ilicifolium* (seaweed)	Water extraction	Betanodavirus	Fishes	Cytopathic effect reduction assay	Significant antiviral activity.	[144]
*Salvia plebeia* R. Br.	Alcohol precipitation	Respiratory syncytial virus (RSV)	mice	In vitro and in vivo antiviral	Fractions (Mw ≥ 10,000 Da) inhibit the RSV proliferation and reduce the lung lesions induced by RSV.	[145]
Red algae	Water extraction	Influenza viruses SARS-CoV-2	Madin–Darby canine kidney cells, African green monkey kidney cells, human embryonic kidney (HEK) 293 T cells, mice.	In vitro and in vivo antiviral	*λ*-carrageenan reduced expression of viral proteins in cell lysates and suppressed progeny virus production. A dose-dependent effect was reported.	[142]
*Asarum* polysaccharides	Water extraction and alcohol precipitation	H1N1 influenza virus	Cell real-time monitoring system and Reed-Muench mice model	In vitro and in vivo antiviral	Good anti-influenza virus activity.	[146]
*Isatidis radix*	Ethanol extraction	Hepatitis B virus (HBV)	HepG2.2.15 cell	In vitro antiviral	No toxicity at <400 μg/mL. Doses of 50, 100, and 200 μg/mL significantly reduced extracellular and intracellular levels of HBsAg, HBeAg, and HBV DNA in HepG2.2.15 cells.	[147]
*Pleurotus pulmonarius*	Water and ethanol extraction	Influenza virus A California/07/09 (H1N1pdm)	The neutral red adsorption test was used in the study	In vitro antiviral	The ethanol extracts exhibit a more potent antiviral effect than that of water extracts. Weak toxicity was reported.	[148]

**Table 4:** Polysaccharide derivatives reported to have antiviral activities.

Modifications	Polysaccharides	Antiviral Effects	Mechanisms	References
Sulfatation	Dextran sulfate	Anti-ZIKV, IAV *, enveloped viruses	Inhibition of adsorption and replication	[153,154,155]
	3-O-Sulfated heparin	Anti-HSV-1	Inhibition of adsorption	[156]
	MI-S, FR-S	Anti HSV-1 *, HSV-2 *	Inhibition of adsorption	[157,158]
	sAAPs, sTPS	Anti-NDV	-	[159,160]
	SPLCf	Anti HSV, PV-1	Inhibition of adsorption and transcription	[161]
	sCVPS	Anti-NDV *	-	[162]
	S1F1, S2F1	Anti HSV-1, HSV-2	-	[163]
	NCMCS	Anti-HIV-1, RLV	Inhibition of virus adsorption	[164]
	*κ*-carrageenan	Anti-IAV *, HIV	Inhibition of replication	[165]
Phosphorylation	pRCPS, pCIPS	Anti-DHAV	Inhibition of replication	[166,167]
	pRCPS	Anti-CPV	-	[168]
Complexes	Chitosan–chondroitin sulfate	Anti-HIV	Inhibition of replication	[169]
Enzymatic modification	Chitooligosaccharides	Anti-HIV-1	Inhibition of adsorption	[170]
Acylation + sulfation	O-acylated carrageenans	Anti-HIV	-	[171]
Aminoderivatized	Aminoethyl-chitosan	Anti-HIV-1	-	[172]

* Inhibitory effect observed in vivo. Sulfated *Auricularia auricula* polysaccharides (sAAPs), sulfated Tremella polysaccharides (sTPS), sulfated polysaccharide of Caesalpinia ferrea (SPLCf), sulfated Chuanmingshen violaceum polysaccharide (sCVPS), *N*-carboxymethylchitosan-N-O-sulfate (NCMCS), phosphorylated Codonopsis pilosula polysaccharide (pCPPS), phosphorylated *Chrysanthemum indicum* polysaccharide (pCIPS), and phosphorylated Radix Cyathulae officinalis Kuan polysaccharides (pRCPS).

## Data Availability

Not applicable.
